# Editorial: Genome editing in stem cells

**DOI:** 10.3389/fgeed.2024.1357369

**Published:** 2024-01-17

**Authors:** Delger Bayarsaikhan, Edina Poletto

**Affiliations:** ^1^ nSAGE, Incheon, Republic of Korea; ^2^ School of Medicine, Stanford University, Stanford, CA, United States

**Keywords:** CRISPR, genome editing, stem cells, CRISPR/Cas9, embryonic stell cell

Over the past decade, genome editing has undergone unprecedented progress with the Clustered Regularly Interspaced Short Palindromic Repeats (CRISPR) system, revolutionizing biomedical research. Many tools were developed using the gRNA/Cas9 complex and its precise target recognition feature, allowing creative applications of this system and its use in multiple settings. In this Research Topic, we present innovative research articles using CRISPR/Cas9 and its derivative tools, as well as their application on stem cells.

Understanding how these genome editing tools work in detail and how we can make them more efficient can shed light on physiological processes unknown so far. We know that the cell cycle phase plays a significant role in the DNA repair process and, consequently, it profoundly affects the editing efficiency, at least regarding the Non-Homologous End Joining (NHEJ) and Homology-Directed Repair (HDR) pathways. Base editors, the next-in-line gene editing tool, are believed to rely less on the cell cycle phase, but this had not been directly tested. By synchronizing cells in the G1 phase, Burnett et al. describe that nickase-derived base editors are active regardless of the cell cycle, while non-nicking base editors (based on dead Cas9) highly rely on the S-phase of the cell cycle. Interestingly, editing C•G to A•T can be significantly increased if performed during the G1 phase, which can be particularly interesting for non-dividing cells. This is the first time the higher efficiency of base editors in post-mitotic cells is directly demonstrated. Moreover, bulk mRNAseq showed that base editing did not perturb the transcription profile of edited cells, not even in the DNA repair pathways, unlike what has been seen with double-stranded break-based strategies, which could be potentially beneficial for the clinical application of base editors.

Exemplifying the vast applicability of CRISPR, Heath et al. developed a creative approach to image unique DNA sequences with high sensitivity and low background signal. Using a split Luciferase biosensor, single base edits were identified in live cells, with clear differentiation between zero and two-allele edits. This new approach provides a faster screening strategy for edited cells than labor-intense single-cell isolation and sequencing, for example. In line with non-orthogonal CRISPR/Cas9 approaches is epigenome modification. By fusing a deactivated Cas9 with chromatin modulators, such as histone acetyltransferases and DNA methyltransferases, one can specifically activate or silence target genes. This is particularly interesting when pharmacological drugs that modulate the epigenome lack specificity and lead to important side effects when used in the clinical setting. Using a deactivated Cas9 fused to p300 histone acetyltransferase, Namous et al. added acetyl groups to the promoter region of the *PRM1* gene, which functions as a DNA compactor in sperm cells and is silenced in other cell types. Upon acetylation and thus activation of *PRM1* with this system, tumor cells had an impactful decrease in cell proliferation. This study shows how genome editing tools can be used to modulate alternative pathways, potentially becoming a therapeutic strategy in cancer treatment.

The final two studies in this Research Topic use CRISPR/Cas9 as a tool to unravel new sequences and comprehend gene function in stem cells. Yu et al. analyzed the Satb1 gene, which maintains trophoblast stemness. They identified multiple transcript variants, promoters, and distant regulatory sequences that control its expression in murine trophoblasts. Likewise, Wang et al. used human trophoblast stem cells to analyze the nuclear morphology and the syncytialization process following the CRISPR/Cas9-mediated disruption of the *LMNA* gene, which is closely involved in nuclear enlargement. Both studies contribute to filling existing gaps in our knowledge of stem cells.

As illustrated in the featured articles, the CRISPR/Cas9 toolkit has expanded beyond its original application in genomic DNA sequence editing, now enabling epigenome modification, gene expression modulation, and real-time detection of DNA or RNA sequences, protein interactions and molecular events in live cells. We have also reached the milestone of having CRISPR being used as a therapy for certain genetic conditions, with promising outcomes.

Aligning CRISPR tools with stem cells holds the potential to develop novel treatments for a wide range of diseases, heralding a new era of precision medicine that could revolutionize healthcare in the future.

